# Continuing professional development training needs of medical laboratory personnel in Botswana

**DOI:** 10.1186/1478-4491-12-46

**Published:** 2014-08-18

**Authors:** Ishmael Kasvosve, Jenny H Ledikwe, Othilia Phumaphi, Mulamuli Mpofu, Robert Nyangah, Modisa S Motswaledi, Robert Martin, Bazghina-werq Semo

**Affiliations:** 1 Department of Medical Laboratory Sciences, University of Botswana, Private Bag UB 00712, Gaborone, Botswana; 2 Botswana International Training and Education Center for Health (I-TECH), P.O. Box AC46 ACH, Riverwalk, Gaborone, Botswana; 3 Department of Global Health, University of Washington, 901 Boren Avenue Suite 1100 Seattle, WA 98104-3508, USA

**Keywords:** Continuing professional development, Curriculum, Developing countries, Medical laboratory, Training needs

## Abstract

**Background:**

Laboratory professionals are expected to maintain their knowledge on the most recent advances in laboratory testing and continuing professional development (CPD) programs can address this expectation. In developing countries, accessing CPD programs is a major challenge for laboratory personnel, partly due to their limited availability. An assessment was conducted among clinical laboratory workforce in Botswana to identify and prioritize CPD training needs as well as preferred modes of CPD delivery.

**Methods:**

A self-administered questionnaire was disseminated to medical laboratory scientists and technicians registered with the Botswana Health Professions Council. Questions were organized into domains of competency related to (i) quality management systems, (ii) technical competence, (iii) laboratory management, leadership, and coaching, and (iv) pathophysiology, data interpretation, and research. Participants were asked to rank their self-perceived training needs using a 3-point scale in order of importance (most, moderate, and least). Furthermore, participants were asked to select any three preferences for delivery formats for the CPD.

**Results:**

Out of 350 questionnaires that were distributed, 275 were completed and returned giving an overall response rate of 79%. The most frequently selected topics for training in rank order according to key themes were (mean, range) (i) quality management systems, most important (79%, 74–84%); (ii) pathophysiology, data interpretation, and research (68%, 52–78%); (iii) technical competence (65%, 44–73%); and (iv) laboratory management, leadership, and coaching (60%, 37–77%). The top three topics selected by the participants were (i) quality systems essentials for medical laboratory, (ii) implementing a quality management system, and (iii) techniques to identify and control sources of error in laboratory procedures. The top three preferred CPD delivery modes, in rank order, were training workshops, hands-on workshops, and internet-based learning. Journal clubs at the workplace was the least preferred method of delivery of CPD credits.

**Conclusions:**

CPD programs to be developed should focus on topics that address quality management systems, case studies, competence assessment, and customer care. The findings from this survey can also inform medical laboratory pre-service education curriculum.

## Background

Health professional boards worldwide are increasingly requiring practitioners to demonstrate their engagement with continuing professional development (CPD) in order to maintain competence in light of the ever-changing scope of practice and technological advances in the medical sciences. The enforcement of this requirement varies from country to country and between professions. The objective of CPD is to maintain high standards of competence in terms of knowledge, skills, and behavior [1,2]. Literature exists indicating that CPD in the health professions is effective in improving healthcare, patient outcomes, and population health. For example, participation in CPD activities by physicians has been shown to improve the quality of care given to patients and the public [3]. Studies have also shown that physicians who engage in CPD are more likely to accept new and effective treatment modalities and discontinue use of existing lower-benefit practices resulting in improved patient outcomes [4].

Health professional boards in some developed countries have embraced CPD as an effective way of maintaining and improving competencies of health professionals and have made them mandatory [5,6]. For example, participation in CPD for medical laboratory scientists is a pre-requisite for a salary adjustment and career advancement in developed countries [7-10]. Health professional boards in developing countries are making progress in establishing and enforcing CPD requirements for re-licensing [11]. In Uganda and South Africa, regulatory bodies require completion of CPD credits in order to re-register [12]. Despite the benefits of CPD engagement on patient outcomes, in some developing countries there are several challenges regarding access to CPD programs [13]. Hindrances to participation in CPD include limited internet facilities to access online CPD programs, infrequent national professional meetings, lack of funding to attend regional and international conferences, and few to non-existent national CPD providers that specifically cater for medical laboratory professionals. In most cases, the only opportunities available for CPD are the non-structured in-house trainings organized by employers.

In Botswana, as in many other developing countries, formal CPD programs to address lifelong learning needs of laboratory professionals are lacking. The Botswana Health Professions Council (BHPC), the licensing board for healthcare workers, now requires medical laboratory scientists and technicians to accumulate CPD credit points as part of their career-long learning and to retain professional registration [14]. Since the focus of CPD is to enhance roles and competencies so as to improve patient outcomes through improved practice, CPD activities should be planned and designed (i) to address the current needs of the laboratory professionals, (ii) for re-registration, and (iii) to foster innovation.

The development of CPD programs must be based on an empirical assessment of needs and planners should focus on addressing shortfalls between existing knowledge or skill and needed competencies [15-17]. Consequently, needs assessment studies should focus on the actual and predicted professional practice requirements, related enabling competence and capabilities, and corresponding learning and change requirements [18]. Studies have shown that the format of CPD is strongly linked to improvement of competence, with educational techniques that are centered on interaction and active participation (e.g., case discussion, role-play, hands-on practice sessions) being more effective than practice guidelines and formal instruction (e.g., didactic lectures, seminars) [19,20]. In the development of an effective CPD program, it is therefore important to understand both the specific needs of the target population, and to investigate appropriate educational formats for CPD delivery.

The primary objective of this needs assessment survey was to identify and prioritize current development needs of medical laboratory scientists and technicians in order to address performance requirements. Secondly, we wanted to identify the format preferences for CPD delivery for medical laboratory scientists and technicians in Botswana.

## Design and methods

### Study design

This was a descriptive cross-sectional assessment utilizing a self-administered questionnaire.

### Study population

A questionnaire was distributed to 350 medical laboratory scientists and technicians registered with the BHPC. A laboratory technician is defined as a professional holding a diploma qualification in medical laboratory technology obtained after three years of post-high school study, and a laboratory scientist holds a bachelor’s degree in medical laboratory sciences. The population surveyed included professionals working in clinical laboratories, medical laboratory supplies businesses, and training and research institutions. Since the target population was finite and could theoretically be accessed at economical costs, the study intended to recruit the entire target population. Informed consent was obtained from the study participants. The protocol was approved by Institutional Review Boards at the University of Botswana and University of Washington. A Research Permit was obtained from the Botswana Ministry of Health.

### Data collection

The questionnaire was developed with input from various stakeholders which included representatives of employers, training institutions, and a professional society for laboratory professionals. Questions were organized into key domains of competency related to (i) quality management systems, (ii) technical competence, (iii) laboratory management, leadership, and coaching, and (iv) pathophysiology, data interpretation, and research. Participants were asked to rank their self-perceived training needs using a 3-point scale in order of importance (most, moderate, and least). Furthermore, participants were asked to select any three preferences for delivery formats for the CPD activities from a list of seven options, namely face-to-face presentations, live video conference, hands-on workshops, journal club at the workplace, webcast of distance lecture/internet-based teaching modules, training workshops, and directed learning in the workplace followed by a quiz.

According to the literature, potential learners themselves are considered the best source of information about their training needs, but this approach alone might not give the full picture of the clinical laboratories’ training priorities [21]. Therefore, to address this limitation, the study also solicited views of participants in supervisory roles (n = 90). The supervisors completed additional questions addressing the training needs of the staff they supervised. The questionnaire was disseminated in-person or via e-mail, with a follow-up telephone call to confirm if the practitioner had received the questionnaire. Furthermore, at every site we selected one practitioner to collect the completed questionnaires and return them by post.

### Data analysis

The data collected by the questionnaire was entered into a spreadsheet and data analysis was performed using SPSS version 19 software. We generated descriptive statistics to characterize the demographics of the respondents. Frequencies were used to report individuals who identified each CPD key theme and topic. We also generated frequencies to report preferences of CPD method of delivery. A χ^2^ test was used to compare training needs between medical laboratory scientists and laboratory technicians; and according to number of years of post-qualification experience (<10 years vs. ≥10 years).

Topics to be included in CPD programs to be developed were prioritized as follows: topics given a most importance rating by ≥80% of the respondents (priority 1); 70 to 79% (priority 2); 60 to 69% (priority 3), and ≤59% (priority 4).

## Results

### Characteristics of the study population

Three hundred and fifty questionnaires were distributed to laboratory scientists and technicians and 275 (79%) responded to the survey, of which 56% were employed as medical laboratory technicians, 32% as medical laboratory scientists, 4% as laboratory managers, and 8% were employed in teaching or research institutions (Table 1). The participants had varying years of service, ranging from less than one year to over 10 years of experience. The majority (85%) were working in a public sector laboratory and the rest were employed in the private sector or a training/research institution. Ninety participants performed supervisory roles at their workplace; 34 (38%) supervised 1 to 4 staff members; while 31 (34%), 10 (11%), and 12 (13%) supervised 5 to 9, 10 to 14, and more than 15 staff members, respectively.

**Table 1:** **Demographic characteristics of participants** (**n** = **275**)

		**Frequency**	**Percentage**
**Cadre type**			
	Medical laboratory technicians	153	55.6
	Medical laboratory scientists	89	32.4
	Laboratory managers	12	4.4
	Others*	21	7.6
**Years of service**			
	<1 year	22	8
	1–5 years	73	26.4
	6–10 years	72	26.4
	>10 years	105	38.0
	Not specified	3	1.1
**Current place of work**			
	Ministry of Health laboratory	234	85.1
	Private laboratory	30	10.9
	Military hospital laboratory	3	1.1
	Educational or research institution	5	2.2
	Not specified	3	0.7
**Supervisory responsibilities**			
	Number reporting supervisory roles	90	33
	Number not reporting supervisory roles	185	67

*These participants were either working for a non-governmental organization or a training institution.

### CPD educational preferences of the laboratory personnel

The most frequently selected topics for training in rank order according to key themes were (i) quality management systems, most important (mean, range 79%, 74% to 84%); (ii) pathophysiology, data interpretation, and research (68%, 52% to 78%); (iii) technical competence (65%, 44% to 73%); and (iv) laboratory management, leadership, and coaching (60%, 37% to 77%) (Table 2). Most medical laboratory professionals in Botswana felt they needed more training on topics in quality management systems. Laboratory management, leadership, and coaching was the least rated skills domain.

**Table 2:** **Ranking of training needs according to pre**-**determined themes**/**domains**, **n** = **275**

**Training needs**	**Ranking**			
	**Most important n (%)**	**Moderate importance n (%)**	**Low importance n (%)**	**No response n (%)**
**Quality management systems**				
Quality system essentials for medical laboratory	230 (84)	20 (7)	8 (3)	17 (6)
Implementing a quality management system	230 (84)	23 (8)	4(2)	18 (7)
Techniques to identify and control sources of errors in laboratory procedures	220 (80)	29 (11)	5 (2)	21 (8)
Management of non-conformances in laboratory services	213 (78)	37 (14)	6 (2)	19 (7)
Use of external quality assessment to improve testing procedures	202 (74)	39 (14)	9 (3)	25 (9)
Internal quality control and Westgard rules	211 (77)	33 (12)	8(3)	23 (8)
Laboratory accreditation: principles and processes	211 (77)	35 (13)	6 (2)	23 (8)
Clinical laboratory safety	209 (76)	35 (13)	10 (4)	21 (8)
**Technical competence**				
Evidence-based laboratory medicine	188 (68)	52 (19)	12 (4)	23 (8)
Evaluation and selection of analytical methods and equipment	193 (70)	54 (20)	9 (3)	19 (7)
Definition, establishment, and use of reference ranges	192 (70)	49 (18)	12 (4)	22 (8)
Health informatics	149 (54)	89 (32)	10 (4)	27 (10)
Point of care testing	122 (44)	99 (36)	32 (12)	22 (8)
Statistics in laboratory medicine	180 (66)	68 (25)	11 (4)	16 (6)
Specimen management	201 (73)	36 (13)	15 (6)	23 (8)
Molecular diagnostic methods and genetics	190 (69)	59 (22)	14 (5)	12 (4)
Laboratory and disease surveillance	186 (68)	62 (23)	5 (2)	22 (8)
Equipment maintenance	176 (64)	50 (18)	16 (6)	33 (12)
**Laboratory management, leadership, and coaching**				
Customer care	211 (77)	34 (12)	11 (4)	19 (7)
Competence assessment	197 (72)	49 (18)	10 (4)	19 (7)
Ethics and professionalism	159 (58)	78 (28)	16 (6)	22 (8)
Supervision and delegation	134 (49)	95 (35)	19 (7)	27 (10)
Preceptorship and mentorship	196 (71)	57 (21)	12 (4)	10 (4)
Data management, report writing, and presentation skills	126 (46)	100 (37)	23 (8)	26 (10)
Basic cost accounting for clinical laboratory services	183 (67)	58 (21)	11 (4)	23 (8)
Management of resources and supplies	185 (67)	56 (20)	10 (4)	24 (9)
Monitoring and evaluation	166 (60)	72 (26)	16 (6)	21 (8)
Team building	188 (68)	54 (20)	12 (4)	21 (8)
Strategic planning	184 (67)	61 (22)	10 (4)	20 (7)
Rational selection of tests	162 (59)	75 (28)	13 (5)	25 (9)
Medical tariffs (billing and coding)	102 (37)	95 (35)	49 (18)	29 (11)
Costing of laboratory tests and procedures	117 (43)	93 (34)	38 (14)	27 (10)
**Pathophysiology, data interpretation, and research**				
Case studies in clinical microbiology	214 (78)	34 (12)	8 (3)	19 (7)
Case studies in clinical chemistry	200 (73)	40 (15)	9 (3)	26 (10)
Case studies in hematology	208 (76)	38(14)	8 (3)	21 (8)
Case studies in medical parasitology	202 (74)	36 (13)	12 (4)	25 (9)
Case studies in blood transfusion science	207 (75)	36 (13)	11 (4)	21 (8)
Case studies in cytology and histology	183 (67)	47 (17)	19 (7)	26 (10)
Research proposal development and operational research	194 (71)	43 (16)	12 (4)	26 (10)
Grant proposal writing	149 (54)	76 (28)	17 (6)	33 (12)
Manuscript preparation	142 (52)	76 (28)	19 (7)	38 (14)
Basic computer skills (Microsoft suite)	160 (58)	47 (17)	35 (13)	33 (12)

Table 3 prioritizes the training topics according to the ranking by the participants; 80% or more of the participants felt they needed training on (i) quality systems essentials in the medical laboratory, (ii) implementing a quality management system, and (iii) techniques to identify sources of error in laboratory procedures.

**Table 3:** Prioritization of topics for training according to ranking by participants

**Priority 1**	**Priority 2**	**Priority 3**	**Priority 4**
**≥ 80%**	**70%-79%**	**60%-69%**	**(≤59%**
Quality system essentials for medical laboratory	Management of non-conformances in laboratory services	Evidence based laboratory medicine	Health informatics
Implementing a quality management system	Use of external quality assessment to improve testing procedures	Statistics in laboratory medicine	Point of care testing
Techniques to identify and control sources of errors in laboratory procedures	Internal quality control and Westgard Rules	Molecular diagnostic methods and genetics	Ethics and professionalism
	Laboratory accreditation: principles and processes	Laboratory and disease surveillance	Supervision and delegation
	Evaluation and selection of analytical methods and equipment	Equipment maintenance	Data management, report writing and presentation skills
	Definition, establishment and use of reference ranges	Basic cost accounting for clinical laboratory services	Rational selection of tests
	Specimen management	Management of resources and supplies	Medical tariffs (Billing and coding)
	Customer care	Monitoring and evaluation	Costing of laboratory tests and procedures
	Competence assessment	Team building	Grant proposal writing
	Preceptorship and mentorship	Strategic planning	Manuscript preparation
	Case studies in clinical microbiology	Case studies in cytology and histology	Basic computer skills (Microsoft suite)
	Case studies in clinical chemistry		
	Case studies in hematology		
	Case studies in medical parasitology		
	Case studies in blood transfusion science		
	Research proposal development and operational research		

The participants indicated the topics as most important.

Overall, the ranking of training needs was comparable between medical laboratory scientists and technicians. There was no difference between the two cadres for the skills domains technical competence and laboratory management, leadership, and coaching. For some topics on pathophysiology, data interpretation, and research, more technicians felt they needed the training compared to medical laboratory scientists. However, the majority (≥56%) in each group selected the same topics for CPD training. Comparison of training needs according to post-qualification experience revealed no marked differences for most topics, but more recently qualified laboratory professionals (≤10 years’ experience) felt they needed training on case studies and competence assessment than those with >10 years’ experience (*P* <0.05).

### Training needs identified by supervisors

Ninety participants holding supervisory positions identified topics on technical competency (24%), and a similar proportion (23%) identified laboratory management, leadership, and coaching as key themes of greatest training need for their staff; 14% of the supervisors felt there was a need to train staff on topics on quality management systems.

### Preferred format for CPD

**Figure 1:**
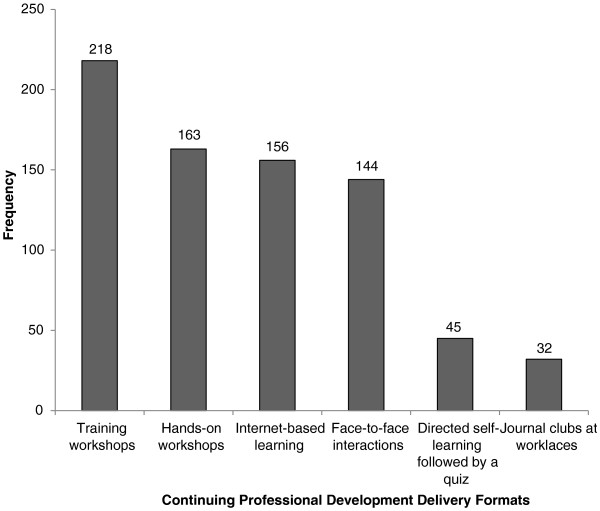
Format preferences for delivery of continuing professional development activities, n = 275.

The top three preferred CPD delivery modes, in rank order, were training workshops (218/275 participants), hands-on workshops (163/275 participants), and internet-based learning (156/275 participants) (Figure 1). A journal club at the workplace was the least preferred method of delivery and was selected by only 32 participants.

## Discussion

CPD is essential in supporting sustained competence of the healthcare workforce. This is the first study to report on CPD needs and educational preferences for medical laboratory personnel in Botswana. Generally, the ranking of training needs by the survey respondents did not vary by qualification or years of experience, with the topics selected being comparable between medical laboratory scientists and laboratory technicians. However, the less experienced personnel felt they needed training on case studies compared to the more experienced staff.

The majority of respondents identified topics on quality management systems, case studies, competence assessment, and customer care as the most important for training. Most of the public sector laboratories in Botswana are in the process of implementing quality management systems [Matema D, Chief Scientific Officer, Ministry of Health, Oral communication, 2014]. Laboratory personnel may feel the need to learn more about quality management systems in order to successfully implement the process in their laboratories; this may be the reason behind the greatest need for training in quality management systems. It is also plausible that training addressing quality management systems in clinical laboratory services has not been fully integrated into pre-service education curricula [22]. A review of the diploma training program curriculum in Botswana conducted in 2007 recommended strengthening of training of quality management systems in pre-service programs [Motswaledi MS, Deputy Director of Clinical Services, Oral communication, 2008]. The identified need for training in laboratory quality management systems is in keeping with reported competence gaps in the laboratory workforce in resource-limited countries [23].

CPD programs on case studies should seek to address disease profiles in the country. Although research proposal development and operational research had a high priority, the related topics “grant proposal writing and manuscript preparation” received a low priority by the respondents.

Participants in supervisory roles were asked to identify learning needs of the staff they supervise. Surprisingly, the topics recommended by supervisors were not congruent with observations from their supervisees. The supervisors believed that CPD programs should focus on technical competence, laboratory management, leadership, and coaching. A possible reason for this variation is that self-assessments are more likely to be influenced by what the professionals think they will gain from a professional growth and future advancement perspective [24,25], while supervisor assessments are likely to be more focused on technical competencies required to improve laboratory testing.

The three most selected platforms of CPD delivery in rank order were training workshops, hands-on workshops, and internet-based learning. Training workshops and hands-on workshops are invariably more expensive than internet-based learning. These approaches may involve travelling and time spent away from work, whereas internet-based learning can be comparatively inexpensive. However, studies have shown that approaches involving interaction with learners are more effective educational techniques [26].

The selection of the CPD delivery platforms may be due to social desirability. Familiarity with the method could also have influenced the choice of methods. Training workshops with laboratory personnel travelling to a central site is a common approach of disseminating knowledge and skills in the country and this might also have influenced the choices selected by the respondents. Further, lack of reliable internet services and inadequate technology awareness could be reasons why most participants did not select internet-based platforms for CPD delivery. However, we did not ask the respondents to indicate comfort levels and access to internet services.

The other limitation to the study is that a single quantitative method was employed to identify CPD training needs of laboratory personnel. Population-based surveys are subject to low response rates and selection bias, thereby reducing the generalizability of the findings [27]. However, a mixed-methods approach (for instance, key informant interviews, and focus groups) to identify CPD training may address these shortcomings. The use of a variety of methods to confirm the same information by different methods or sources can increase the validity of the findings. In this study, 79% of the target population participated in the survey. Another potential limitation of the study was response bias. Since development and implementation of CPD programs in Botswana may be perceived by participants as being beneficial to their professional growth and addressing requirements for re-registration with the regulatory body, this may have motivated the target population to participate in the study.

The information on suggested topics and the form of delivery collected in this survey may be helpful in planning future CPD programs for medical laboratory workers in Botswana and the region. The findings from this survey can also inform medical laboratory pre-service education curriculum as well as focus the specific contribution that development partners may wish to render.

## Conclusions

CPD programs should focus on topics that address quality management systems, competence assessment, customer care, and case studies on disease profiles common in Botswana. The three most preferred CPD delivery platforms in rank order were training workshops, hands-on workshops, and internet-based learning. However, when implementing CPD programs, providers need to determine the most effective and sustainable method for delivery of CPD content. While the findings from this survey provide a baseline data for medical laboratory science CPD program development, CPD providers should also consider future trends in laboratory medicine and advances in technology. Medical laboratory science pre-service education programs should also strengthen components of quality management systems in their curricula. Overall, it is noted that CPD needs will vary over time, depending on the prevailing challenges of the practice. This underscores the need for continuous evaluation of practice needs to remain relevant.

## Abbreviations

BHPC: Botswana Health Professions Council; CPD: Continuing professional development.

## Competing interests

The authors declare that they have no competing interests.

## Authors’ contributions

IK and RN conceived the study and design; IK, JL, OP, MM, and BS participated in the implementation of the study and data analysis. IK wrote the first draft and JL and BS provided general oversight in implementing the study. All the authors participated in the write up, review, and approval of the manuscript.
